# Positive crosstalk between EGFR and the TF-PAR2 pathway mediates resistance to cisplatin and poor survival in cervical cancer

**DOI:** 10.18632/oncotarget.25748

**Published:** 2018-07-17

**Authors:** Vitor Hugo de Almeida, Isabella dos Santos Guimarães, Lucas R. Almendra, Araci M.R. Rondon, Tatiana M. Tilli, Andréia C. de Melo, Cinthya Sternberg, Robson Q. Monteiro

**Affiliations:** ^1^ Instituto de Bioquímica Médica Leopoldo De Meis, Universidade Federal do Rio de Janeiro, Rio de Janeiro, RJ, Brazil; ^2^ Divisão de Pesquisa Clínica e Desenvolvimento Tecnológico, Instituto Nacional de Câncer, Rio de Janeiro, RJ, Brazil; ^3^ Centro de Desenvolvimento Tecnológico em Saúde, Fundação Oswaldo Cruz, Rio de Janeiro, RJ, Brazil; ^4^ Present address: Sociedade Brasileira de Oncologia Clínica (SBOC), Belo Horizonte, MG, Brazil

**Keywords:** cervical cancer, epidermal growth factor receptor (EGFR), tissue factor (TF), protease-activated receptor 2 (PAR-2), cyclooxygenase 2 (COX-2)

## Abstract

Cisplatin-based chemoradiation is the standard treatment for cervical cancer, but chemosensitizing strategies are needed to improve patient survival. *EGFR* (*Epidermal Growth Factor Receptor*) is an oncogene overexpressed in cervical cancer that is involved in chemoresistance. Recent studies showed that EGFR upregulates multiple elements of the coagulation cascade, including tissue factor (TF) and the protease-activated receptors (PAR) 1 and 2. Moreover, many G protein-coupled receptors, including PARs, have been implicated in EGFR transactivation. However, the role of coagulation proteins in the progression of cervical cancer has been poorly investigated. Herein we employed cervical cancer cell lines and The Cancer Genome Atlas (TCGA) database to evaluate the role of EGFR, TF and PAR2 in chemoresistance. The SLIGKL-NH2 peptide (PAR2-AP) and coagulation factor VIIa (FVIIa) were used as PAR2 agonists, while cetuximab was used to inhibit EGFR. The more aggressive cell line CASKI showed higher expression levels of EGFR, TF and PAR2 than that of C33A. PAR2 transactivated EGFR, which further upregulated cyclooxygenase-2 (COX2) expression. PAR2-AP decreased cisplatin-induced apoptosis through an EGFR- and COX2-dependent mechanism. Furthermore, treatment of CASKI cells with EGF upregulated TF expression, while treatment with cetuximab decreased the TF protein levels. The RNA-seq data from 309 TCGA samples showed a strong positive correlation between EGFR and TF expression (*P* = 0.0003). In addition, the increased expression of EGFR, PAR2 or COX2 in cervical cancer patients was significantly correlated with poor overall survival. Taken together, our results suggest that EGFR and COX2 are effectors of the TF/FVIIa/PAR2 signaling pathway, promoting chemoresistance.

## INTRODUCTION

Cervical cancer is a public health problem representing the fourth most common cancer in women worldwide, with more than 500,000 new cases per year [1]. Combined treatment involving cisplatin-based chemotherapy and radiotherapy has been established as the standard therapeutic approach for patients with locally advanced disease [2–5]. However, patients with stage III and IV tumors have 5-year survival rates lower than 50% [5], and novel strategies to improve the prognosis of these patients are needed.

Cancer progression depends largely on the activity of cell surface membrane receptors that control several intracellular signal transduction pathways. The epidermal growth factor receptor (EGFR) plays an important role in the process of carcinogenesis and is of prognostic and therapeutic relevance in several cancer types [6]. EGFR belongs to the HER tyrosine-kinase receptor family, and EGFR activation by ligands leads to its dimerization, autophosphorylation and activation of downstream signaling pathways that regulate cell transformation [7]. Previous reports have shown that EGFR is expressed in 80% of cervical carcinomas and is correlated with disease progression [8, 9]. Studies in other models have also revealed that EGFR can be transactivated by several extracellular stimuli, unrelated to EGFR ligands, such as agonists of the G protein-coupled receptors (GPCRs) [10, 11].

EGFR drives robust upregulation of tissue factor (TF) in tumor cells derived from glioblastoma [12]. TF is a 47-kDa transmembrane protein that acts as the high-affinity receptor for coagulation factor VII/VIIa (FVII/FVIIa), leading to the proteolytic activation of coagulation zymogens, such as factor X (FX) and prothrombin, and resulting in the formation of activated proteases and fibrin clot [13]. To date, few studies have investigated the biological role of TF in cervical cancer. Cocco and colleagues analyzed the expression of TF in cell lines as well as in malignant tissues derived from a small cohort of cervical cancer patients. Interestingly, TF expression was observed in 8 out of 8 of the tumor tissues and in 11 out of 11 of the cervical carcinoma cell lines but not in normal cervical keratinocytes [14]. In addition, it has been demonstrated that TF expression is regulated by hypoxia in HPV-infected cervical cancer cells [15].

It is well known that blood coagulation proteases regulate cell functions through specific GPCRs, the protease-activated receptors (PARs) [13]. PARs consist of a family of receptors (PAR1, PAR2, PAR3 and PAR4) that are uniquely activated by proteolytic cleavage of their extracellular portion. This cleavage unmasks a new N-terminus, which serves as a tethered ligand that binds to the second extracellular domain of the protein, resulting in various cellular responses [13]. Of the four mammalian PARs, PAR1, PAR3 and PAR4 can be activated by thrombin, whereas PAR2 can be activated by TF-FVIIa complex but not thrombin [16]. Moreover, the TF-FVIIa-FXa complex may cleave and activate PAR1, PAR2 and PAR4 [16].

PAR1 and PAR2 are usually overexpressed in various human cancer types, and many studies have shown a strong correlation between their expression and aggressive behavior of tumor cells [17–19]. PAR activation has been reported to be coupled to Ca^2+^ mobilization and activation of the mitogen-activated protein kinase (MAPK) pathway [16, 20]. Furthermore, previous studies have shown that PAR1 and PAR2 can induce the transactivation of EGFR in transformed cells [21, 22]. However, the role of PAR-mediated EGFR transactivation in cervical cancer cells has not been previously investigated.

In the current study, we report that EGFR is transactivated by PAR2 agonists in cervical cancer cells, promoting resistance to cisplatin through a COX2-dependent mechanism. EGFR activation also upregulates TF expression. In cervical cancer patients, PAR2, as well as EGFR and COX2, was independently associated with poor prognosis. Our data suggest that the TF-FVIIa-PAR2 signaling axis cooperates with EGFR, revealing the presence of a positive feedback loop that favors tumor progression.

## RESULTS

### EGFR, TF and PAR2 are differentially expressed in cervical cancer cell lines

In the current study, we employed two cell lines derived from cervical squamous cell carcinoma. The C33A cell line does not exhibit HPV infection [23], while CASKI cells are HPV-16 positive [23], derived from a metastatic site and more resistant to cisplatin than C33A cells [24]. Therefore, the CASKI cell line is more aggressive than C33A cells and constitutes an interesting pre-clinical model that represents the vast majority of cervical cancer patients.

**Figure 1:**
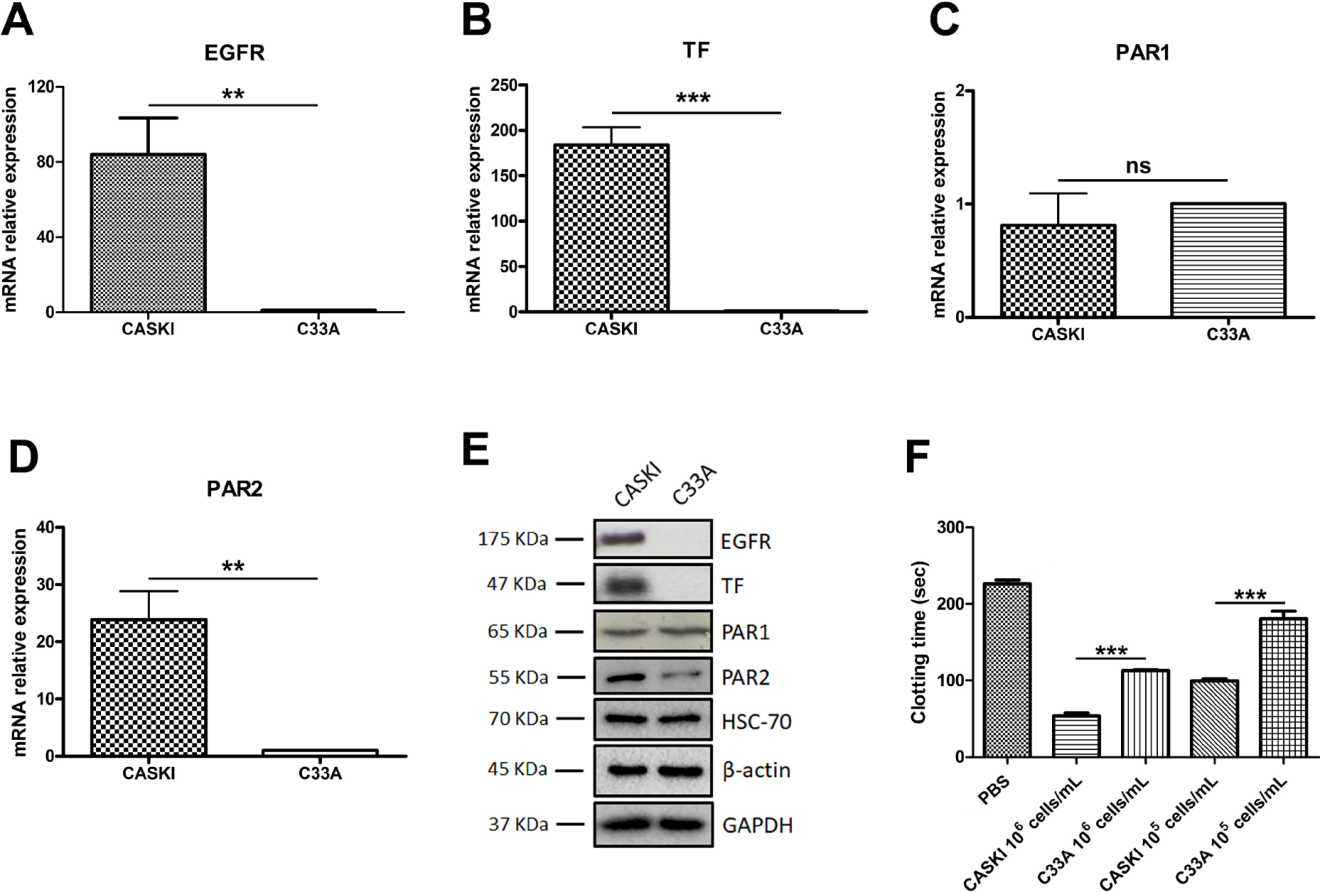
Expression of EGFR, TF, PAR1 and PAR2 in cervical cancer cell lines Total RNA was extracted, and mRNA was converted into cDNA. Gene expression assays for (**A**) *EGFR* (*n* = 4), (**B**) TF (*F3* gene; *n* = 3), (**C**) PAR1 (*F2R* gene; *n* = 4) and (**D**) PAR2 (*F2RL1* gene; *n* = 4) were performed by quantitative PCR. *GAPDH* was used as a reference gene for normalization. The relative expression level of mRNA was calculated using the ΔΔCT method. Values represent mean + SD; ns: not significant, ^**^P < 0.01, ^***^P < 0.001 (two-tailed unpaired *t* test). (**E**) Cells were lysed, and the protein levels of EGFR, TF, PAR1 and PAR2 were determined by Western blotting. HSC70, GAPDH and β-actin were used as loading controls. Representative image from three experiments. (**F**) Clotting time of platelet-poor plasma incubated with cells (ranging from 1 × 10^5^ cells/mL to 1 × 10^6^ cells/mL). The reaction was initiated with CaCl_2_, and the control group (PBS) was evaluated without cells. Values represent mean + SD of four independent experiments; ^**^P < 0.01, ^***^P < 0.001 (one-way ANOVA).

Quantitative PCR (qPCR) analysis showed that CASKI cells strongly express EGFR (Figure 1A), TF (Figure 1B) and PAR2 (Figure 1D) at the mRNA level, whereas the C33A cell line showed low expression levels of these genes. No difference was observed in the mRNA expression of PAR1 between these two cell lines (Figure 1C). These results were further confirmed by Western blotting assays, as shown in Figure 1E. Indeed, EGFR, TF and PAR2 are differentially expressed in CASKI and C33A cell lines.

Expression of TF by tumor cells has been directly associated with procoagulant activity [25, 26]. To test the biological significance of TF expression in cervical cancer cells, we performed plasma clotting assays (Figure 1F). In accordance with its higher TF expression, CASKI cells accelerated the coagulation time of human plasma by 2-fold in relation to C33A. In order to confirm the participation of TF on plasma clotting promotion, CASKI and C33A cells were previously incubated with a neutralizing anti-TF antibody (50 μg/mL). Acceleration of plasma clotting time by CASKI cells was suppressed upon incubation with anti-TF (Supplementary Figure 1).

### EGFR transactivation by PAR2 agonists

**Figure 2:**
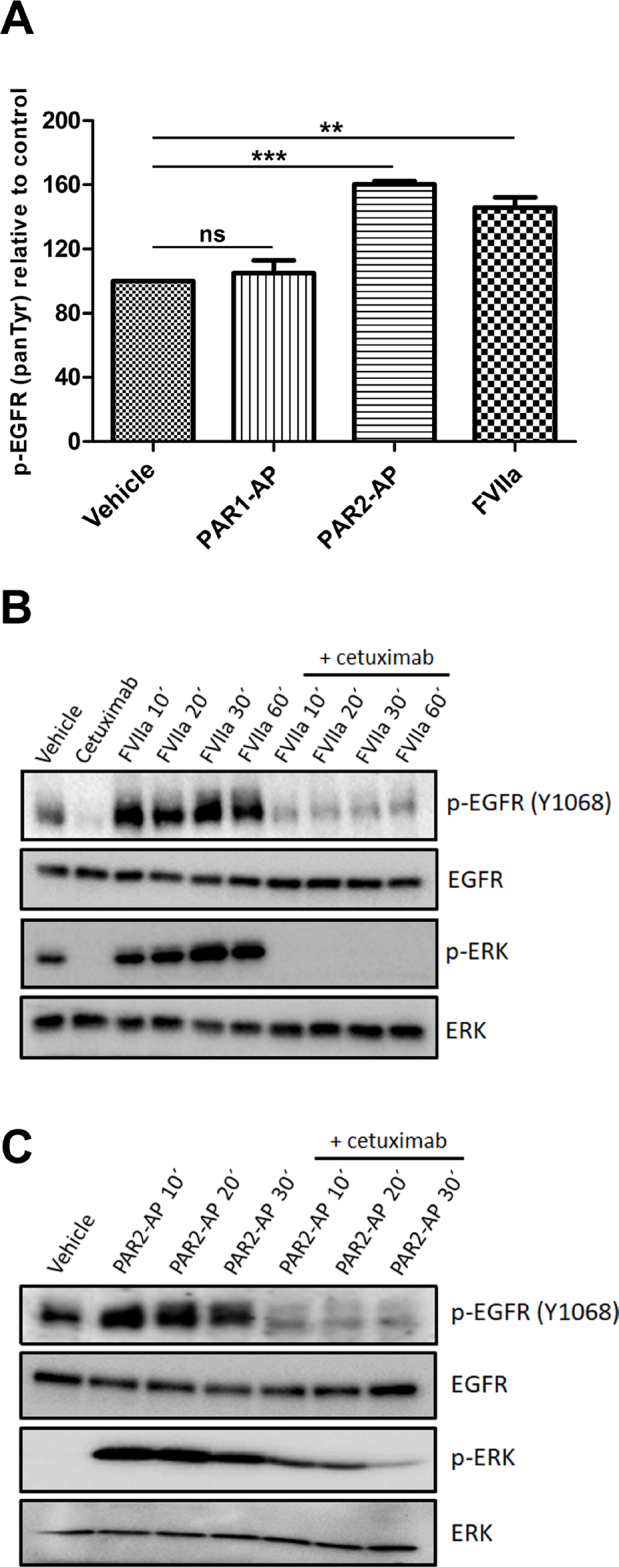
PAR2 transactivates the EGFR-ERK signaling pathway in CASKI cells (**A**) CASKI cells were starved for 16 h followed by stimulation with PAR1-AP (50 µM), PAR2-AP (50 µM) or FVIIa (20 nM) for 15 minutes. After cell lysis, equal amounts of protein were used for the determination of p-EGFR using the PathScan Phospho-EGF Receptor (panTyr) Sandwich ELISA Kit. Values represent mean + SD of three independent experiments; ns: not significant, ^**^P < 0.01, ^***^P < 0.001 (one-way ANOVA). (**B** and **C**) CASKI cells were starved in serum-free medium for 16 h, followed by treatment with cetuximab (100 µg/mL). One hour later, cells were stimulated with FVIIa (20 nM) or PAR2-AP (50 µM) for 10 to 60 minutes. After the incubation time, cells were lysed, and the levels of p-EGFR (Tyr 1068), EGFR, p-ERK 1/2 (Thr202/ Tyr204) and ERK were determined by Western blotting. Representative image from three experiments.

Activation of G protein-coupled receptors has been associated with the transactivation of EGFR [10]. In this context, we examined whether PAR1 or PAR2 transactivated EGFR in cervical cancer cells. CASKI cells were incubated with PAR1- or PAR2-specific agonist peptides (PAR1-AP and PAR2-AP, respectively) or FVIIa, and EGFR phosphorylation (panTyr) was assessed. Activation of PAR2, but not PAR1, caused a significant increase in EGFR tyrosine phosphorylation (Figure 2A). FVIIa, a physiological agonist of PAR2, induced EGFR transactivation to a similar extent as that observed with PAR2-AP (Figure 2A).

Phosphorylation of EGFR at Tyr1068 is a crucial event for downstream protumoral effects, including activation of the ERK/MAPK signaling pathway [27]. As seen in the EGFR phosphorylation (panTyr) assay, treatment of CASKI cells with either FVIIa (Figure 2B) or PAR2-AP (Figure 2C) increased EGFR phosphorylation at Tyr 1068 as well as ERK1/2 activity. The activation of EGFR and ERK1/2 induced by PAR2 was considerably reduced by cetuximab, a monoclonal antibody that binds to EGFR, inhibiting its activation (Figure 2B and 2C). Thus, EGFR transactivation induced by PAR2 agonists contributes to ERK activation in cervical cancer cells.

### EGFR transactivation by PAR2 protects cervical cancer cells against cisplatin-induced apoptosis

Preclinical data from our group showed that the EGFR signaling pathway mediates resistance to chemoradiation in cervical cancer cells [24]. Thus, we next examined the contribution of PAR2-induced EGFR transactivation to chemoresistance.

**Figure 3:**
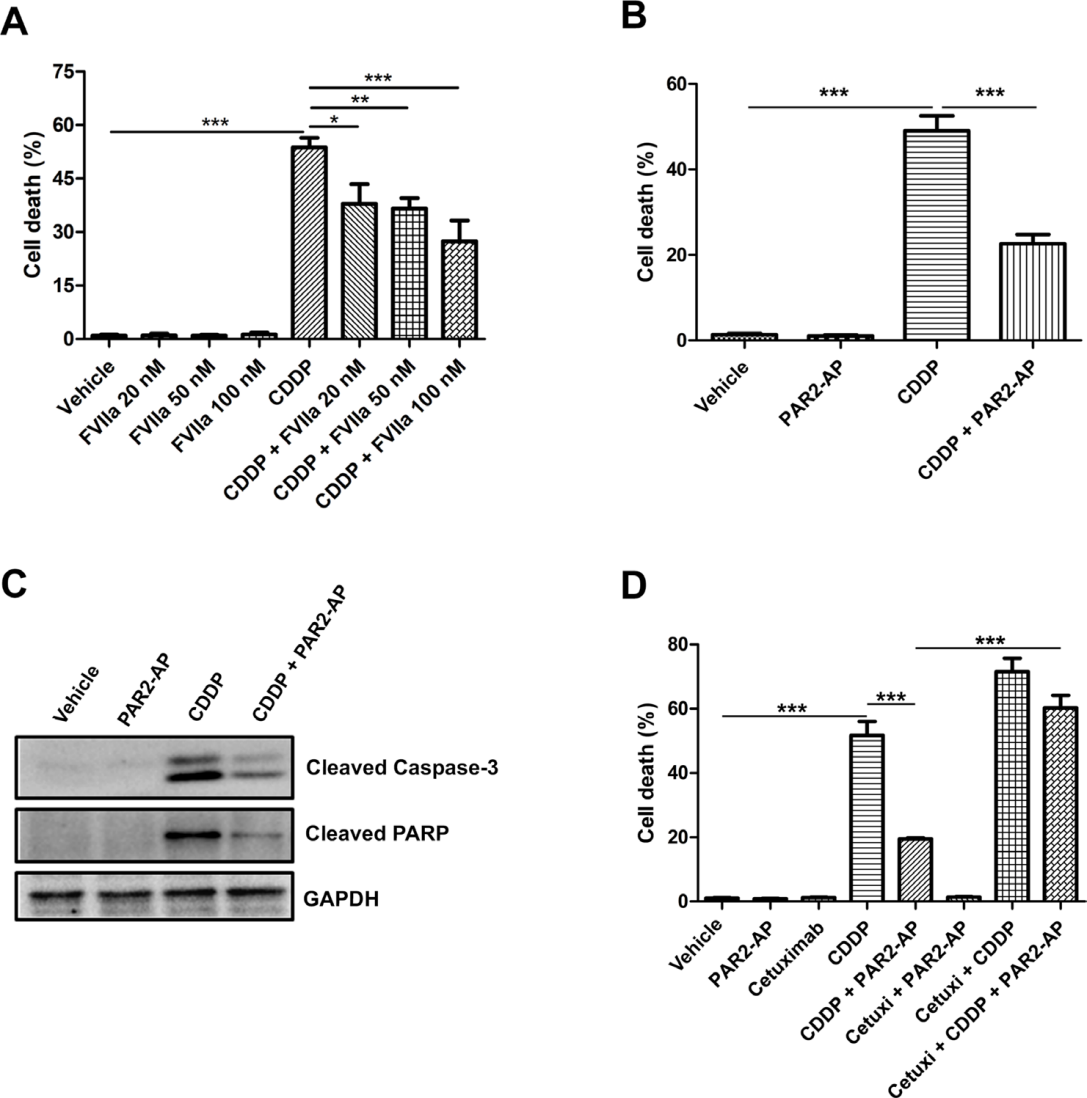
PAR2 activation protects CASKI cells against cisplatin-induced apoptosis through an EGFR-dependent mechanism CASKI cells were starved in serum-free medium for 4 h, followed by treatment with cetuximab (100 µg/mL) when indicated. One hour later, cells were stimulated with FVIIa (20 nM, 50 nM or 100 nM) or PAR2-AP (50 µM) for 60 minutes. Next, cells were treated with cisplatin (CDDP, 20 µM) for 48 h. (**A**) FVIIa-mediated chemoresistance was evaluated by propidium iodide staining, followed by flow cytometry. Cells with fragmented DNA (sub-G1 peak) were considered apoptotic cells. Values represent mean + SD of three independent experiments; ^*^P < 0.05, ^**^P < 0.01, ^***^P < 0.001 (one-way ANOVA). (**B**) PAR2-AP-mediated chemoresistance was evaluated by propidium iodide staining, followed by flow cytometry. Values represent mean + SD of six independent experiments; ^***^P < 0.001 (one-way ANOVA). (**C**) PAR2-AP-mediated chemoresistance was also evaluated by western blot for cleaved caspase-3 and cleaved PARP. GAPDH was used as a loading control. Representative image from three experiments. (**D**) Cetuximab reversed the resistance to cisplatin promoted by the PAR2 activation. Apoptosis was evaluated by propidium iodide staining, followed by flow cytometry. Values represent mean + SD of three independent experiments; ^***^P < 0.001 (one-way ANOVA).

CASKI cells were stimulated with FVIIa (Figure 3A) or PAR2-AP (Figure 3B) and were further treated with cisplatin for 48 h, when apoptosis was evaluated. Internucleosomal DNA fragmentation is a key feature of apoptosis [28], occurring in a caspase-3-dependent manner [29]. The CASKI cell line treated with cisplatin exhibited a high proportion of cells with sub-G1 DNA content (∼50%, Figure 3A and 3B), as shown by flow cytometry, indicative of cells undergoing DNA fragmentation. PAR2 activation with FVIIa significantly decreased the percentage of cells displaying this apoptotic marker in a dose response manner (Figure 3A). As seen with FVIIa, PAR2-AP induced chemoresistance, protecting CASKI cells against cisplatin-induced apoptosis (∼25%, Figure 3B). Moreover, cisplatin induced the cleavage of caspase-3 and PARP (Figure 3C). On the other hand, pretreating CASKI cells with PAR2-AP decreased cleavage of caspase-3 and PARP induced by cisplatin (Figure 3C). Caspase-3 is a critical executioner of apoptosis requiring proteolytic processing of its inactive zymogen into activated p17 and p12 fragments [28], as observed by Western blotting. PARP, a 116-kDa nuclear poly (ADP-ribose) polymerase, is one of the main cleavage targets of caspase-3 *in vivo*, serving as a marker of cells undergoing apoptosis [28].

To prove that PAR2-induced cisplatin resistance is dependent on EGFR transactivation, CASKI cells were pretreated with cetuximab (1 h before PAR2-AP and 2 h before cisplatin). After 48 h, the sub-G1 apoptosis assay was performed. The combination of cetuximab, PAR2-AP and cisplatin increased the percentage of apoptotic cells (∼60%) compared with the combination of PAR2-AP and cisplatin (∼20%) and induced apoptosis to a similar extent observed with cisplatin alone (∼50%) (Figure 3D). Therefore, EGFR inhibition reversed chemoresistance promoted by PAR2 activation in the CASKI cell line.

### PAR2 upregulates cyclooxygenase-2 (COX2) through an EGFR-dependent mechanism

The main mechanisms that lead to clinical resistance to cisplatin have not been fully elucidated. The mechanisms identified so far include increased drug efflux, DNA repair, apoptosis defects and upregulation of cell survival genes [30]. To understand how PAR2 activation promotes chemoresistance in CASKI cells, we evaluated the expression of six genes involved with cisplatin resistance: *ATP7A*, *ATP7B*, *XIAP*, *BAX*, *ERCC1* and *PTGS2* (COX2). ATP7A and ATP7B are copper-transporting ATPases that regulate the level of cisplatin in cells, promoting platinum efflux [31]. ERCC1 is required for the repair of DNA damage caused by many chemotherapeutics, including cisplatin [30]. BAX and XIAP are important apoptosis-regulatory proteins [30], and cyclooxygenase-2 (COX2) is an inflammatory enzyme whose activity can inhibit the apoptosis of tumor cells [32, 33]. Thus, alterations in the expression of these genes could reduce the response of malignant cells to cytotoxic therapy.

**Figure 4:**
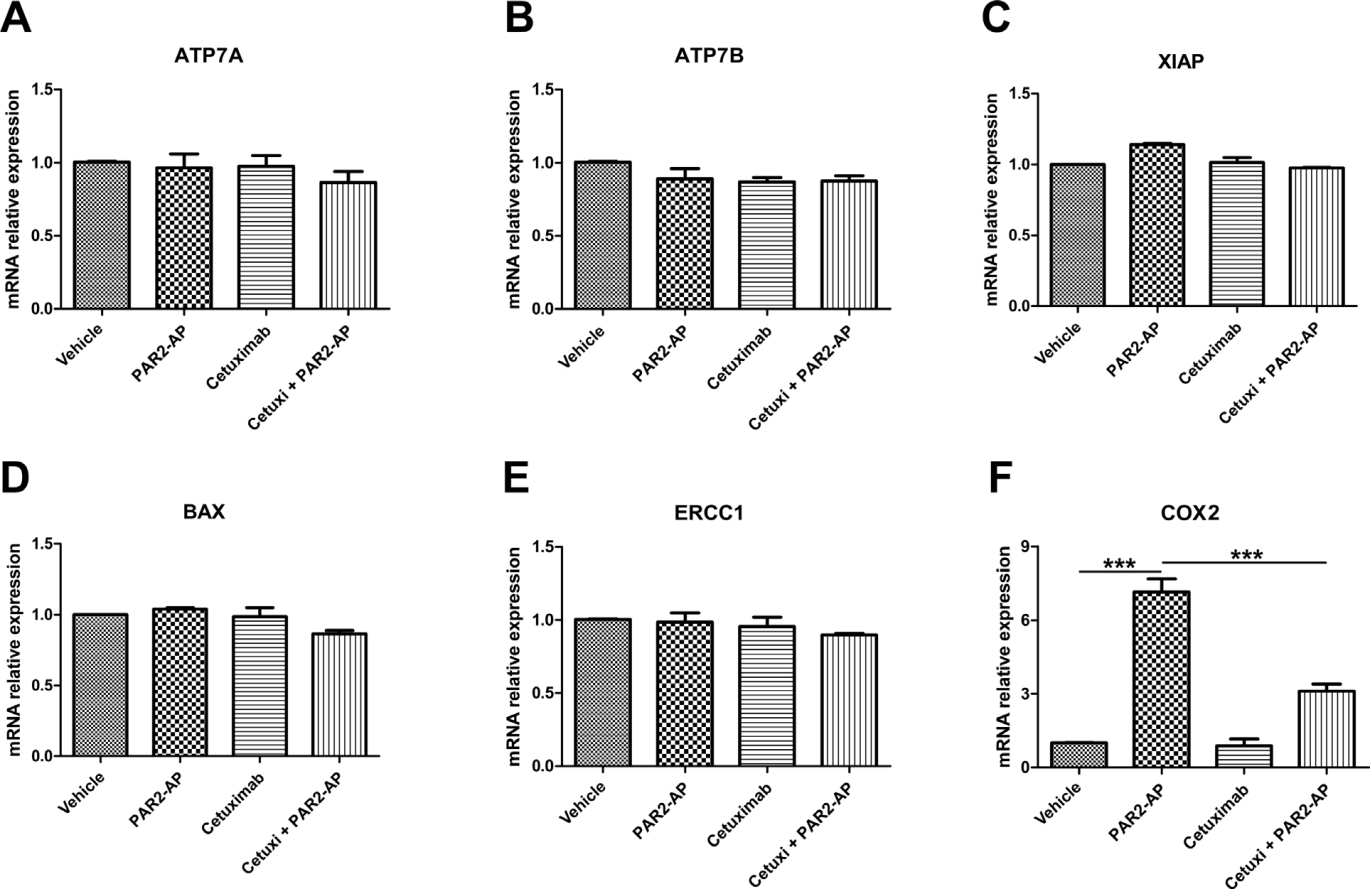
PAR2 activation upregulates cyclooxygenase-2 (COX2) expression through an EGFR-dependent mechanism in CASKI cells Cells were starved for 16 h and were treated with cetuximab (100 µg/mL). One hour later, cells were stimulated with PAR2-AP (50 μM). After 1.5 h, total RNA was extracted, and mRNA was converted into cDNA. The expression of 6 genes involved in the resistance to cisplatin was performed by qPCR: (**A**) *ATP7A*, (**B**) *ATP7B*, (**C**) *XIAP*, (**D**) *BAX*, (**E**) *ERCC1* and (**F**) COX2 (*PTGS2* gene). *GAPDH* was used as a reference gene. The relative expression level of mRNA was calculated using the ΔΔCT method. Values represent mean + SD of four independent experiments; ^***^P < 0.001 (one-way ANOVA).

Treatment of CASKI cells with PAR2-AP did not modulate the expression of ATP7A (Figure 4A), ATP7B (Figure 4B), XIAP (Figure 4C), BAX (Figure 4D) and ERCC1 (Figure 4E) at the mRNA level. On the other hand, PAR2-AP substantially upregulated COX2 expression to approximately 7-fold compared with that of cells treated with vehicle (Figure 4F). As expected, EGFR inhibition by cetuximab blocked COX2 upregulation promoted by PAR2 activation (Figure 4F). Interestingly, weak modulation of COX2 expression was observed in CASKI cells treated with PAR1-AP compared with that of the cells treated with PAR2-AP (Supplementary Figure 2). These data are in accordance with the result of the absence of EGFR phosphorylation promoted by PAR1-AP (Figure 2A). Similarly to PAR2-AP, FVIIa upregulated COX2 expression through an EGFR-dependent mechanism in CASKI cells (Supplementary Figure 3).

### COX inhibition impairs PAR2-mediated chemoresistance

**Figure 5:**
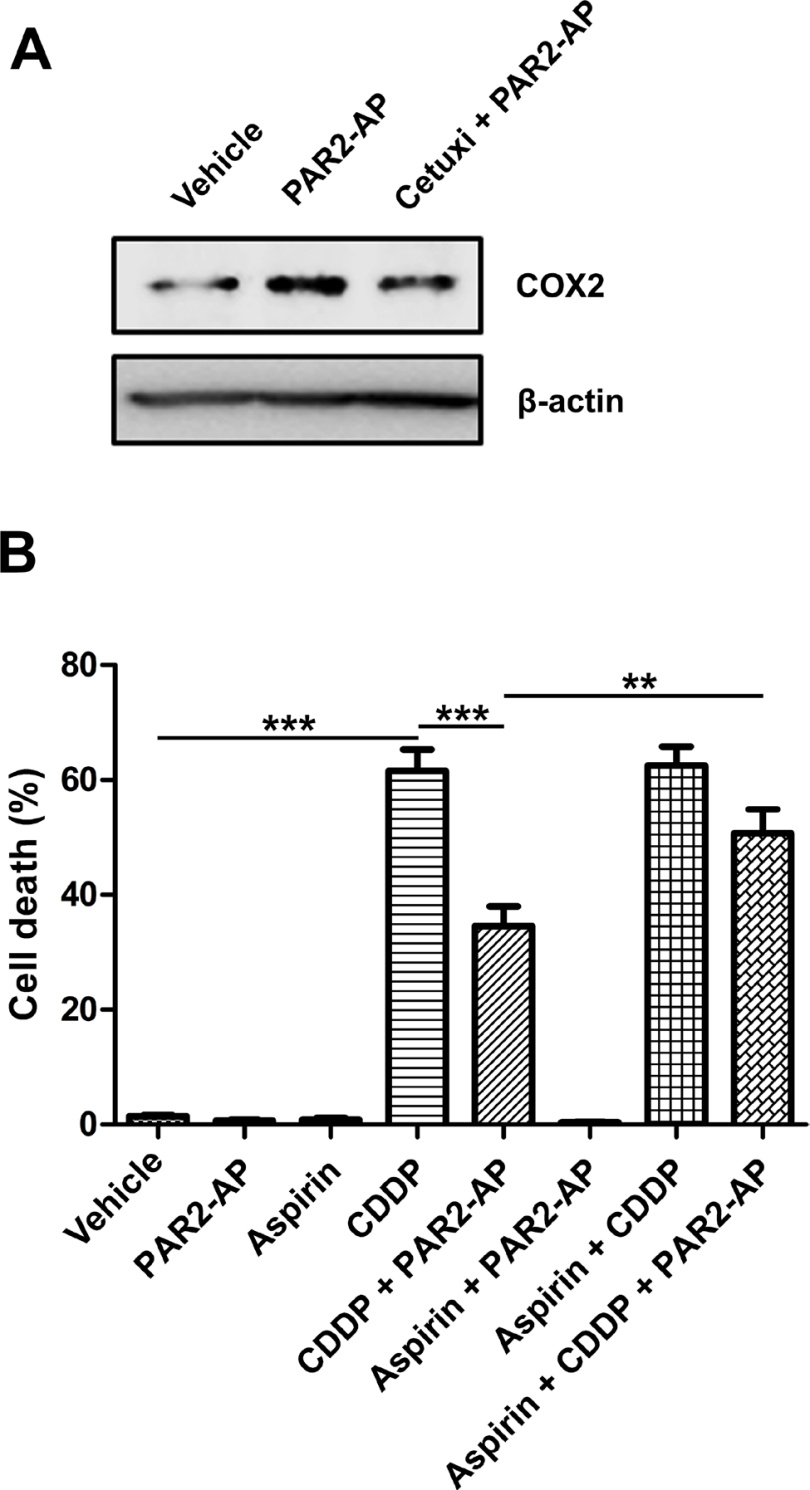
COX inhibition by aspirin impairs chemoresistance promoted by PAR2 activation in CASKI cells (**A**) Cells were starved for 16 h and were treated with cetuximab (100 µg/mL). One hour later, cells were stimulated with PAR2-AP (50 μM). After 3 h, cells were lysed, and the levels of COX2 and β-actin (loading control) were determined by Western blotting (representative image from three experiments). (**B**) CASKI cells were starved in serum-free medium for 4 h, followed by treatment with aspirin (1 mM). One hour later, cells were stimulated with PAR2-AP (50 µM) for 60 minutes. Next, cells were treated with cisplatin (CDDP, 20 µM) for 48 h. Apoptosis was evaluated by propidium iodide staining followed by flow cytometry. Cells with fragmented DNA (sub-G1 peak) were considered apoptotic cells. Values represent mean + SD of four independent experiments; ^**^P < 0.01, ^***^P < 0.001 (one-way ANOVA).

PAR2-AP also induced COX2 upregulation at protein level in CASKI cells (approximately 2-fold relative to cells treated with vehicle; Figure 5A). This effect was partially blocked by pretreatment with cetuximab (1.2-fold relative to vehicle; Figure 5A).

To evaluate the role of COX2 as an effector of the TF-PAR2-EGFR pathway in cisplatin resistance, CASKI cells were pretreated with aspirin (acetylsalicylic acid) 1 h prior to PAR2-AP stimulation and 2 h before cisplatin. After 48 h, apoptosis was evaluated by the sub-G1 assay. Aspirin is a nonsteroidal anti-inflammatory drug that exerts its effect primarily by interfering with the biosynthesis of prostanoids generated by cyclooxygenase-1 and -2 enzymes. Aspirin impairs prostanoids synthesis through the inhibition of COX by irreversible acetylation of serine 530 [34].

Cisplatin induced 62% of apoptosis in CASKI cells, while prestimulation with PAR2-AP, one hour before exposure to cisplatin, decreased the rate of apoptotic cells to 34% (Figure 5B). The combination of aspirin, PAR2-AP and cisplatin induced 51% of apoptosis, preventing resistance to cisplatin mediated by the PAR2 agonist (Figure 5B). Interestingly, aspirin at lower doses, capable of inhibiting COX1 activity without interfering with COX2 activity, did not reverse the chemoresistance promoted by PAR2 activation (data not shown). Our results suggest the involvement of COX2 in the TF-FVIIa-PAR2-EGFR signaling pathway and in the chemoresistance of cervical cancer cells.

### EGFR upregulates TF expression *in vitro* and correlates with TF expression in cervical cancer samples

**Figure 6:**
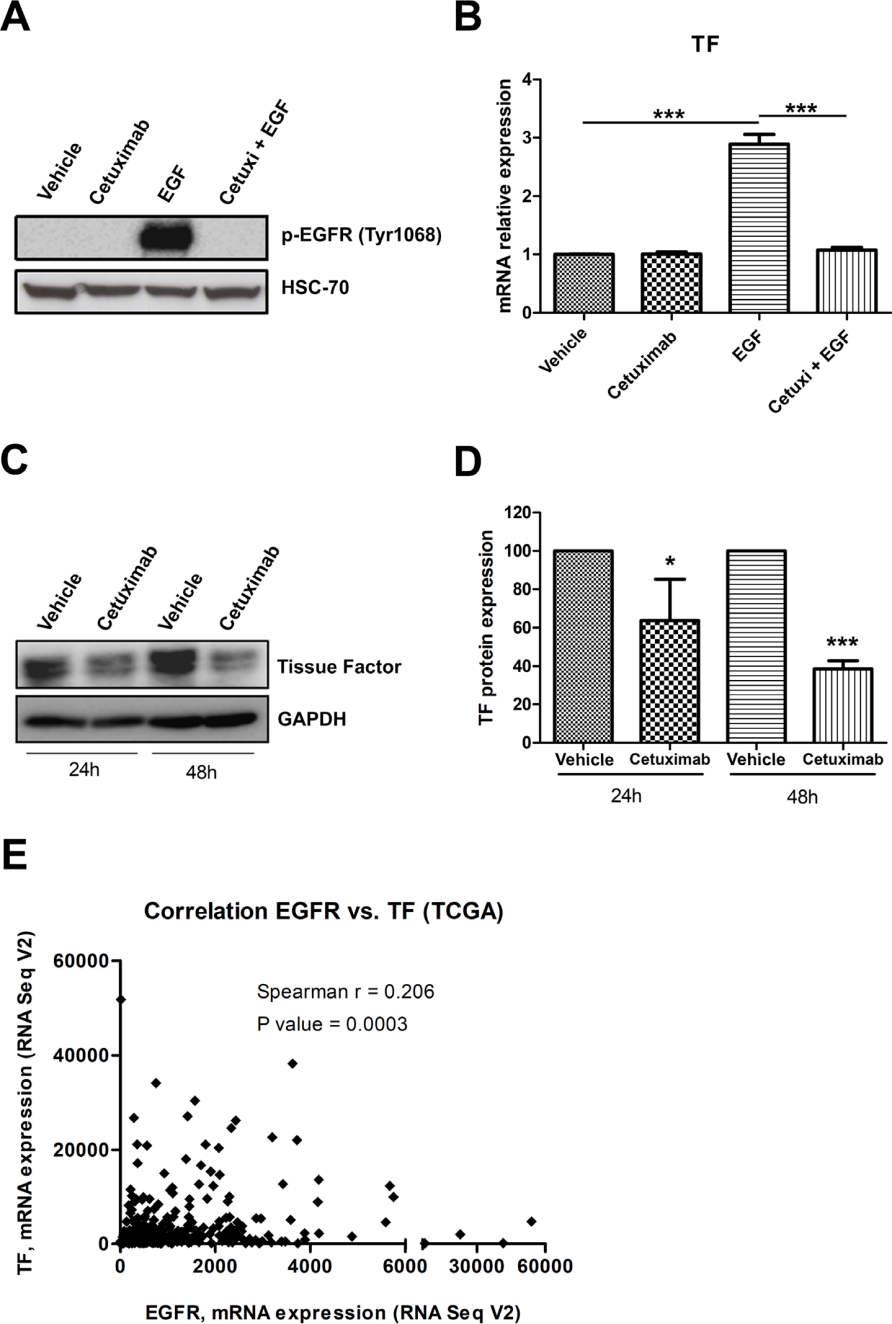
EGFR activation upregulates TF in cervical cancer cells (**A**) CASKI cells were starved for 16 h and were treated with cetuximab (100 µg/mL). One hour later, cells were stimulated with EGF (50 ng/mL). After 10 min, cells were lysed, and the levels of p-EGFR (Tyr 1068) and HSC70 (loading control) were determined by Western blotting (representative image from three experiments). (**B**) After starving for 16 h, CASKI cells were treated with cetuximab (100 µg/mL). One hour later, cells were incubated with EGF (50 ng/mL). After treatment for 1.5 h, total RNA was extracted, and mRNA was converted into cDNA. Gene expression assay for TF (*F3* gene) was performed by quantitative PCR. *GAPDH* was used as a housekeeping gene. The relative expression level of mRNA was calculated using the ΔΔCT method. Values represent mean + SD of four independent experiments; ^***^P < 0.001 (one-way ANOVA). (**C**) CASKI cells were starved for 4 h followed by treatment with cetuximab (100 µg/mL) for 24 h or 48 h. Next, cells were lysed, and the levels of TF and GAPDH (loading control) were determined by Western blotting (representative image from three experiments). (**D**) Densitometric analysis of TF and GAPDH bands using ImageJ software (NIH, USA). Values represent mean + SD of three independent experiments; ^*^P < 0.05 and ^***^P < 0.001 (two-tailed unpaired *t* test). (**E**) Gene expression correlation analysis between EGFR and TF in 309 cervical cancer samples. The RNA-seq data were obtained from TCGA, and the correlation was analyzed by Spearman’s test.

Recent studies have demonstrated that EGFR activation drives TF upregulation in tumor cells derived from glioblastoma and vulvar cancer [12, 35]. To directly test whether EGFR modulates TF expression in cervical cancer, we treated CASKI cells with cetuximab one hour before recombinant epidermal growth factor (EGF). As expected, treatment with EGF for 10 minutes increased EGFR phosphorylation at Tyr 1068, and cetuximab completely inhibited this effect (Figure 6A). EGF treatment for 90 minutes increased TF expression at mRNA level by 3-fold compared with that of the cells treated with vehicle (Figure 6B). Cetuximab entirely blocked EGF-induced TF upregulation (Figure 6B). As reported in Figure 1E, CASKI cells showed high TF expression levels under basal conditions. Interestingly, prolonged treatment of CASKI cells with cetuximab for 24 h or 48 h decreased TF expression, at protein level, by approximately 35% and 60% respectively (Figure 6C and 6D). Consistent with our *in vitro* results, we found a significant positive correlation between the mRNA expression levels of EGFR and TF in transcriptome data from cervical cancer patients deposited in The Cancer Genome Atlas - TCGA (Spearman’s *r* = 0.206, *P* = 0.0003) (Figure 6E). Therefore, our results suggest that EGFR upregulates TF expression *in vitro* and in malignant tissues derived from cervical cancer.

### EGFR, PAR2 and COX2 correlate with poor prognosis in cervical cancer

The data from 307 patients with cervical cancer were deposited in TCGA. The median age was 46 years (range: 20–88 years). Regarding the histological subtype, 82.2% of the patients were diagnosed with cervical squamous cell carcinoma. However, the overall survival data are available for 290 patients. In the criteria established in our study, the mRNA upregulation of *EGFR*, TF (*F3* gene), PAR1 (*F2R* gene), PAR2 (*F2RL1* gene) and COX2 (*PTGS2* gene) was found in 12%, 6%, 7%, 7% and 4% of the cases, respectively.

**Figure 7:**
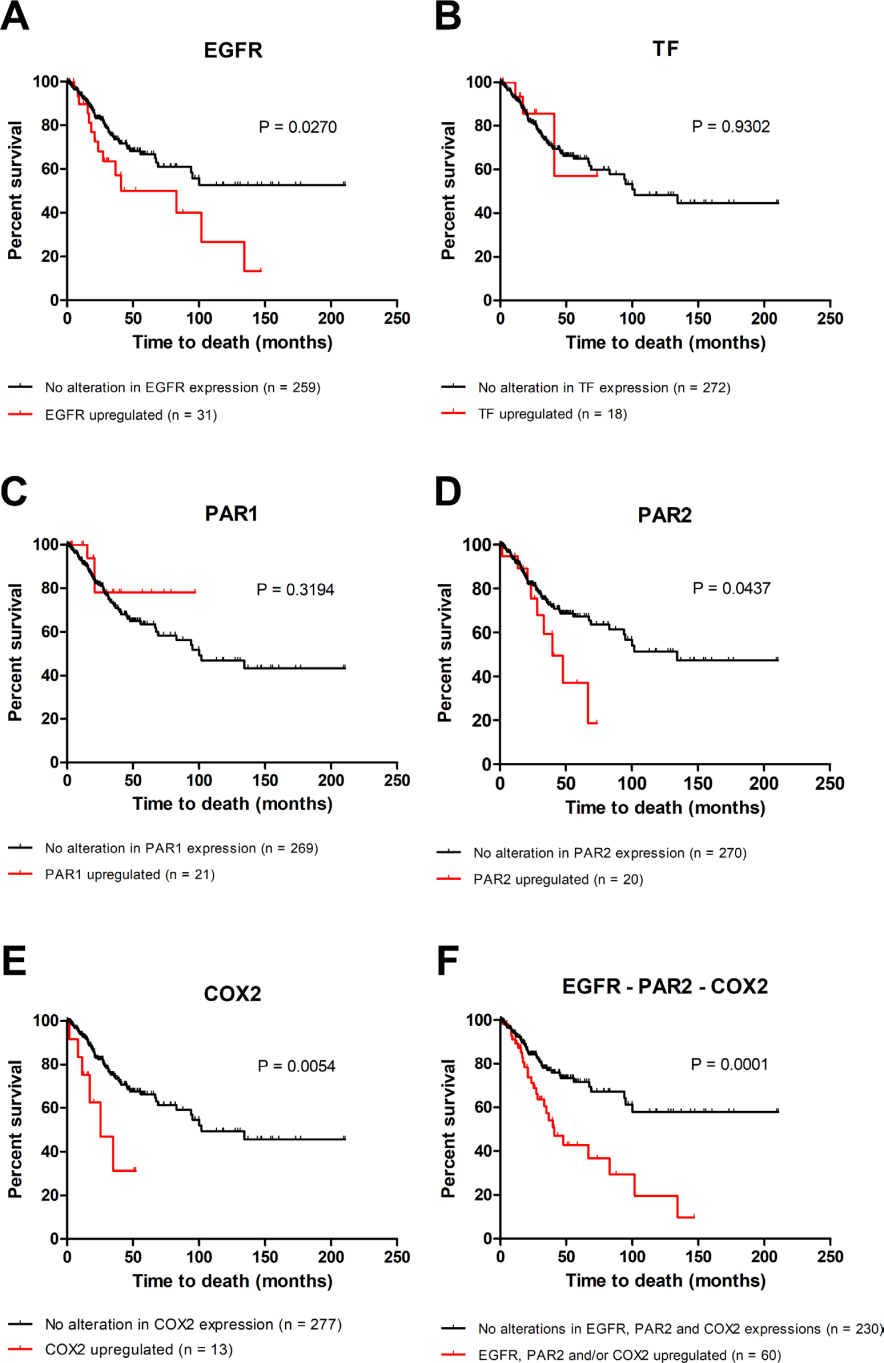
Upregulation of EGFR, PAR2 and COX2 is associated with poor overall survival in cervical cancer Using cBioPortal, overall survival studies were performed to evaluate the relationship between the upregulations of EGFR (**A**), TF (**B**), PAR1 (**C**), PAR2 (**D**) and COX2 (**E**) and prognosis in cervical cancer (TCGA, provisional; *n* = 290). The combined effect of the upregulation of EGFR and/or PAR2 and/or COX2 (in other words, patients with upregulation in one, two or all three genes) on overall survival was also analyzed (**F**). The Kaplan–Meier method was used to construct survival curves, and statistical significance among these curves was determined by the log-rank test.

As shown in Figure 7, there were not significant correlations between the overall survival and gene expression alterations of TF (Figure 7B) and PAR1 (Figure 7C). On the other hand, upregulation of EGFR (*P* = 0.027, Figure 7A), PAR2 (*P* = 0.0437, Figure 7D) or COX2 (*P* = 0.0054, Figure 7E) showed independent prognostic value.

We further analyzed the effect of the combined upregulation of EGFR and/or PAR2 and/or COX2 (patients with upregulation in one, two or all three genes) and this group of patients (*n* = 60) was significantly associated with poor overall survival (median survival = 40.9 months; Figure 7F). Interestingly, the median survival in the group of patients without upregulation in either EGFR, PAR2 and COX2 (*n* = 230) was not reached even after 210 months of follow-up, suggesting a potent protective effect when none of the 3 genes is upregulated (Figure 7F; *P* = 0.0001). Therefore, the combined effect of the upregulation of EGFR and/or PAR2 and/or COX2 showed higher prognostic value than each of the isolated genes in the clinical outcome, reinforcing the presence of a positive crosstalk between PAR2 and the EGFR signaling pathway, which mediates COX2 induction, chemoresistance and worse survival in the cervical cancer model.

## DISCUSSION

Locally advanced cervical cancer treatment has been based on cisplatin and radiation therapy since 1999 [2–4] and, after almost 20 years, few advances have been made in the treatment of this type of cancer. The failure to respond to chemoradiation is a major concern in cervical cancer patients. To this end, studies to detect the molecular mechanisms underlying chemoresistance are in high demand. Identification of signaling pathways implicated in the response of tumor cells to chemotherapy may allow the prediction of treatment outcome and offer novel chemosensitizing strategies through employment of selective inhibitors for these pathways.

EGFR is largely associated with cisplatin resistance in several tumor models [36–38], including ours as showed in Figure 3C, since EGFR inhibition sensitized CASKI cells to cisplatin-induced cell death. One pathway leading to EGFR signaling that has not been thoroughly investigated in cervical cancer progression involves transactivation by G-protein-coupled receptors, such as PAR2 [10, 11, 22]. Accumulated evidences have suggested two mechanisms for the transactivation of EGFR. In the first mechanism, GPCR stimulation leads to the activation of a metalloprotease, resulting in the proteolytic processing of membrane-bound EGFR ligands. Subsequent release of the mature growth factor into the extracellular medium activates the EGFR in an autocrine/paracrine manner [11, 39]. Alternatively, EGFR is transactivated by GPCR without detectable EGF-like ligands, suggesting that EGFR transactivation by GPCR occurs through intracellular signaling pathways. This ligand-independent mechanism involves the activation of intracellular protein tyrosine-kinases, such as Src family proteins. The increased Src activity mediates the phosphorylation of EGFR in its cytosolic domain [39].

In the current study, we demonstrate that PAR2 agonists (PAR2-AP and FVIIa) transactivate EGFR, culminating in the ERK/MAPK phosphorylation. Treatment with cetuximab blocked EGFR-ERK signaling pathway activation mediated by PAR2 agonists. Cetuximab is one of the most successful targeted drugs for cancer treatment, already integrated into the therapy of colorectal and head and neck cancers [40]. Because cetuximab inhibits EGFR activation by high-affinity binding to the extracellular domain of the receptor, thus preventing ligand binding [41], our results suggest that EGFR transactivation by PAR2 agonists in CASKI cells occurs predominantly by the shedding of EGFR ligands.

Our results strongly suggest an important role for PAR2 in the chemoresistance of cervical cancer cells, promoting apoptosis evasion. These findings are consistent with a previous study showing that PAR2 decreases apoptosis induced by proinflammatory cytokines in intestinal epithelial cells [42]. Sánchez-Hernández *et al.* [43] showed that PAR2 activation induced the proliferation of cervical cancer cell lines. In the same study, PAR2 expression was evaluated in a small cohort of 16 patients, and all tumors from cervical cancer patients expressed PAR2 [43]. Furthermore, PAR2 expression has been implicated in lymph node metastasis of cervical cancers [44].

In our model, EGFR transactivation by PAR2 induced COX2 expression. Interestingly, Kulkarni *et al.* demonstrated that COX2 is overexpressed in cervical cancer tissues but was undetectable in normal cervical tissue [45]. They also suggested that the mechanism by which COX2 is upregulated in cervical cancer is EGFR-dependent [45]. Moreover, Kim *et al.* [46] showed that the coexpression of EGFR and COX2 may be used as a potent molecular risk factor to predict the poor survival of patients with squamous cell carcinoma of the uterine cervix.

Prostaglandin E2 (PGE2) has been identified as the major COX2-derived prostanoid in tumor cells. PGE2 signals through four pharmacologically distinct GPCRs, EP1, EP2, EP3, and EP4, each of which activates different downstream signaling pathways [47]. Several mechanisms for the suppression of apoptosis mediated by the COX2/PGE2 pathway have been suggested, and engagement of one or more of the EP receptors may be responsible for this pro-survival response [47]. Further studies indicated that the mechanism by which COX2/PGE2 might suppress apoptosis involves the upregulation of the anti-apoptotic protein Bcl-2 via activation of the MAPK/ERK pathway [48]. More recently, several studies have indicated that PGE2 might alter the apoptotic threshold by engaging many pro-survival pathways, including EGFR transactivation [47, 49]. Indeed, increased COX2 expression is associated with chemotherapy resistance and poor survival in cervical cancer patients [50].

It is now widely recognized that a strong correlation exists between cancer and aberrant hemostasis. Patients with various types of cancers often develop thrombosis, a phenomenon commonly referred as Trousseau syndrome [13]. Reciprocally, components of the coagulation cascade also influence cancer progression. The primary initiator of coagulation, TF, has gained considerable attention as a determinant of tumor progression. On complex formation with its ligand, FVIIa, TF influences PAR-dependent tumor cell behavior [13]. TF expression in tumor cells is the result of well-defined upstream events that occur during the process of oncogenic transformation [51], being an important risk factor associated with venous thromboembolism [52]. In colorectal cancer, mutations of both *KRAS* and *TP53* (with loss of p53 function) enhance TF expression [53]. A constitutively active mutant form of EGFR (EGFRvIII) has also been shown to upregulate TF expression in glioblastoma cells [12]. Herein, we observed that cervical cancer cells display enhanced TF mRNA levels upon activation of EGFR. Alternatively, chronic inhibition of EGFR by cetuximab decreased TF protein levels in cultured cells. We also showed a strong correlation between EGFR and TF expression levels in 309 TCGA samples of cervical cancer. Together, these studies, along with our data, suggest that an accumulation of alterations in oncogenes and tumor suppressor genes upregulates TF in tumor cells.

**Figure 8:**
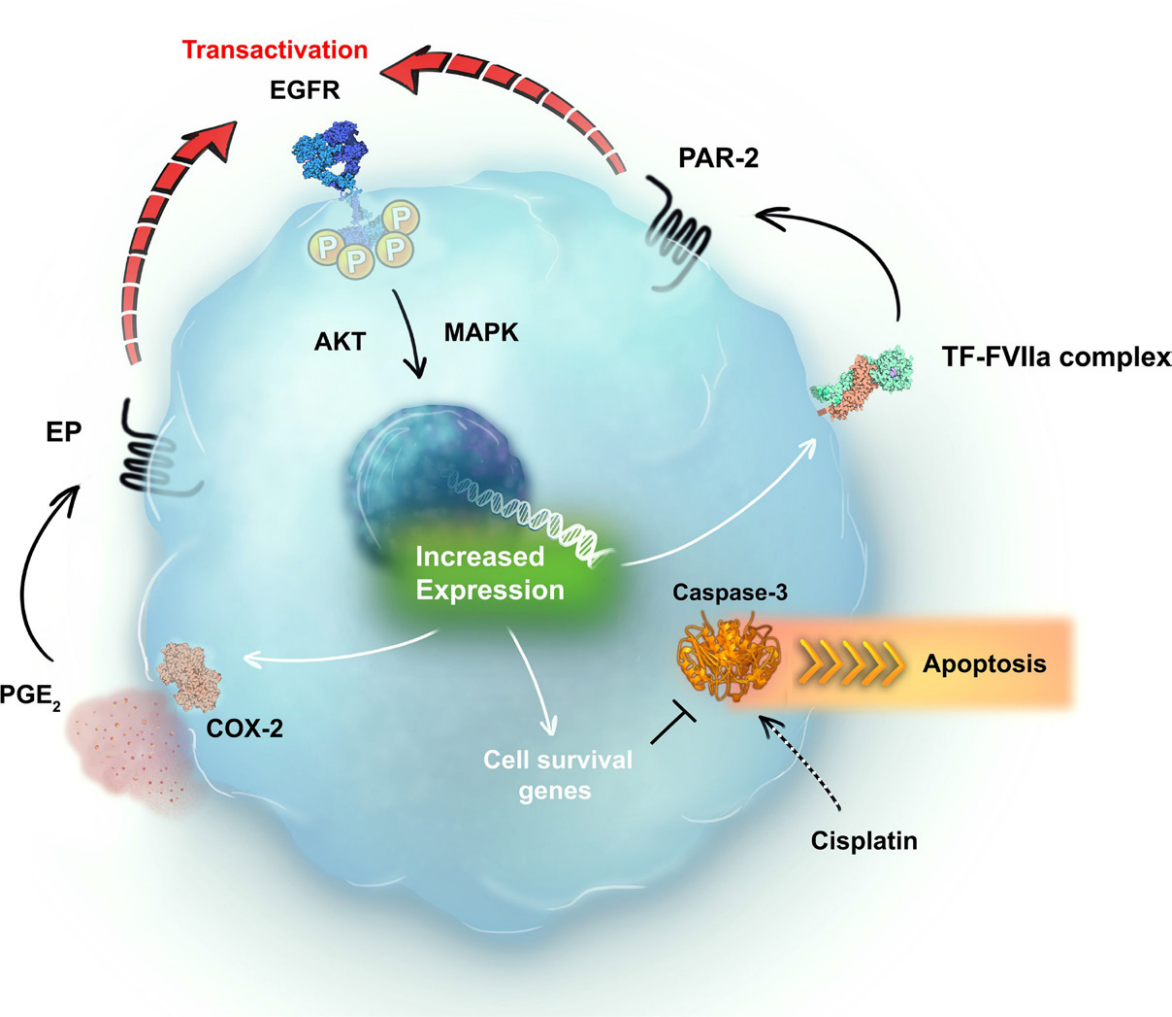
Schematic representation of the positive crosstalk between EGFR and the TF-FVIIa-PAR2 pathway in cervical cancer cells EGFR is transactivated by PAR2 agonists, and EGFR activation increases TF expression. TF-FVIIa binary complex mediates the activation of PAR2, revealing the presence of a positive feedback loop. EGFR transactivation by PAR2 agonists upregulates COX2 expression. COX2-derived PGE2 also transactivates EGFR in several models, suggesting a second positive feedback. In this way, EGFR may be the central pillar of different signaling circuits promoted by GPCRs, inducing chemoresistance. Thus, EGFR, PAR2 and COX2 emerge as novel targets for the treatment of cervical cancer.

Our results demonstrate that PAR2 transactivates EGFR in cervical cancer cells. EGFR activation upregulates TF expression, which, in turn, binds to plasma-derived FVII/FVIIa. On tumor cells, the TF-FVIIa binary complex cleaves and activates PAR2, revealing the presence of a positive feedback loop. At the same time, EGFR transactivation by PAR2 upregulates COX2 expression. The final product of the COX2 enzyme in tumor cells is a prostanoid known as PGE2, which has autocrine and paracrine effects [47]. PGE2 transactivates EGFR through G protein-coupled receptors termed EP [49], which could create a second positive feedback loop. Finally, activation of these signaling pathways decreases cisplatin-induced caspase-3 cleavage in cervical cancer cells, culminating in apoptosis evasion and chemoresistance (Figure 8). Furthermore, EGFR, PAR2 and COX2 have independent prognostic value in cervical cancer patients. Observations such as these merit further investigation of the potential of nonsteroidal anti-inflammatory drugs, EGFR inhibitors and PAR2 antagonists as adjuvants to chemotherapy regimens for patients with cervical cancer.

Precision medicine for cancer treatment is already a reality with the recent introduction of many targeted therapies, however molecular diagnostic tests are needed to identify the patients most likely to benefit from treatment. Indeed, of the 290 patients evaluated in the survival study, only 60 (21%) presented upregulation of EGFR and/or PAR2 and/or COX2 (Figure 7F). Hopefully, the advanced omics technologies will enable identification of aberrant signaling pathways that will greatly facilitate the selection of drugs likely to benefit specific patients [54].

## MATERIALS AND METHODS

### Cell lines

CASKI and C33A cells were cultured in RPMI 1640 medium (Thermo Fisher Scientific, MA, USA) supplemented with 10% fetal bovine serum and incubated in a humidified atmosphere at 37° C in 5% CO_2_. In addition, cells were tested periodically for mycoplasma contamination by the Mycosensor PCR assay (Agilent, CA, USA).

### Chemicals

Cetuximab (monoclonal antibody anti-EGFR) and cisplatin (cis-diamminedichloroplatinum, CDDP) were provided by the Brazilian National Cancer Institute (Rio de Janeiro, Brazil). Monoclonal antibody anti-TF (5G9) was kindly provided by Dr. Wolfram Ruf (Johannes Gutenberg University Medical Center, Mainz, Germany; and Department of Immunology and Microbiology, The Scripps Research Institute, La Jolla, CA). PAR2-specific agonist peptide (PAR2-AP, SLIGKL-NH2) and PAR1-specific agonist peptide (PAR1-AP, TFLLR-NH2) were synthesized by Pepmic Co. LTD, China. Aspirin (acetylsalicylic acid) and human EGF were obtained from Merck, Germany. Human FVIIa was purchased from Haematologic Technologies (VT, USA).

### Gene expression analysis by quantitative PCR

Cells were starved in serum-free medium for 16 h followed by treatment with cetuximab (100 µg/mL) when indicated. One hour later, cells were stimulated with PAR1-AP (50 µM), PAR2-AP (50 µM), FVIIa (20 nM or 50 nM) or EGF (50 ng/mL). After 90 minutes, total RNA was extracted using TRIzol Reagent (Thermo Fisher Scientific). From each sample, 1.0 µg of total RNA was reverse transcribed to cDNA. Next, quantitative PCR was performed on cDNA aliquots using Taqman Fast Real-Time PCR Master Mix (Thermo Fisher Scientific). The gene expression profile was evaluated using the StepOnePlus Real-Time PCR System (Thermo Fisher Scientific). The Taqman gene expression assay references were Hs01076078_m1 (*EGFR*), Hs01076029_m1 (TF, *F3* gene), Hs00169258_m1 (PAR1, *F2R* gene), Hs00608346_m1 (PAR2, *F2RL1* gene), Hs00745222_s1 (*XIAP*), Hs00180269_m1 (*BAX*), Hs00163707_m1 (*ATP7A*), Hs01075310_m1 (*ATP7B*), Hs00157415_m1 (*ERCC1*), Hs00153133_m1 (COX2, *PTGS2* gene) and 4326317E (*GAPDH*). The 2^−ΔΔCT^ method was utilized to analyze the fold increase.

### Western blotting

In experiments analyzing the phosphorylation of signaling pathways, cells were starved in serum-free medium for 16 h followed by treatment with cetuximab (100 µg/mL). One hour later, cells were stimulated with PAR2-AP (50 µM), FVIIa (20 nM) or EGF (50 ng/mL) for 10 to 60 minutes when indicated. To analyze apoptosis, cells were starved for 4 h followed by stimulation with PAR2-AP (50 µM). One hour later, cells were treated with cisplatin (CDDP, 20 µM) for 48 h. In experiments to evaluate COX2 protein expression, cells were starved for 16 h followed by treatment with cetuximab (100 µg/mL). One hour later, cells were stimulated with PAR2-AP (50 µM) for 3 h. Additionally, to evaluate TF downregulation by EGFR inhibition, cells were starved for 4 h followed by treatment with cetuximab (100 µg/mL) for 24 h or 48 h.

After the indicated treatments, cells were lysed, and 30–50 µg of proteins from each sample were run on 6–12% SDS–PAGE and were transferred to a PVDF Hybond-P membrane (GE Healthcare, SP, Brazil). Membranes were incubated with the following antibodies against: EGFR (1:500; #2232, Cell Signaling Technology, MA, USA), TF (1:1000; #4503, American Diagnostica, CT, USA), PAR1 (1:400; sc-13503, Santa Cruz Biotechnology, TX, USA), PAR2 (1:1000; ab180953, Abcam, MA, USA), phospho-p44/42 MAPK (Erk1/2) (1:1000; #9101, Cell Signaling Technology), p44/42 MAPK (Erk1/2) (1:1000; #9102, Cell Signaling Technology), phospho-EGFR Tyr1068 (1:500; #2234, Cell Signaling Technology), cleaved caspase-3 (1:500; #9664, Cell Signaling Technology), cleaved PARP (1:500; #5625, Cell Signaling Technology), COX2 (1:500; #4842, Cell Signaling Technology), HSC70 (1:1000; sc-1059, Santa Cruz Biotechnology), GAPDH (1:1000; #2118, Cell Signaling Technology) and β-actin (1:1000; #8457, Cell Signaling Technology). For analysis of the cleaved caspase-3 expression, membranes were incubated with 1% glutaraldehyde for 30 minutes before blocking [55]. After incubation with the secondary antibodies, immunoblots were detected using the ECL prime reagent (GE Healthcare, SP, Brazil).

### *In vitro* plasma coagulation

Plasma coagulation was measured using a KC-4 Delta coagulometer (Tcoag, Ireland). Blood was collected from healthy donors into 0.105 M sodium citrate vacutainers, and platelet-poor plasma (PPP) was obtained by centrifuging whole blood at 1,000 × *g* for 10 min. Fifty microliters of cells (ranging from 1 × 10^5^ cells/mL to 1 × 10^6^ cells/mL) resuspended in PBS were added to 50 μL of PPP. After incubation at 37°C for 1 minute, 100 μL of 25 mM CaCl2 were added to the cuvettes to initiate plasma clotting. When the anti-TF neutralizing antibody was used, cells (1 × 10^5^ cells/mL) were pre-incubated with the antibody (5G9; 50 μg/mL) for 15 min, at room temperature.

### Enzyme-linked immunosorbent assay (ELISA) for Phospho-EGFR (panTyr)

CASKI cells were starved for 16 h followed by stimulation with PAR1-AP (50 µM), PAR2-AP (50 µM) or FVIIa (20 nM) for 15 minutes. After cell lysis, an equal amount of proteins (300 µg) was used for the determination of p-EGFR using the PathScan Phospho-EGF Receptor (panTyr) Sandwich ELISA Kit (#7911, Cell Signaling Technology) according to the manufacturer’s instructions. Values for receptor phosphorylation were determined by measuring the absorbance at 450 nm.

### Flow cytometry-based apoptosis detection

CASKI cells were starved in serum-free medium for 4 h followed by treatment with cetuximab (100 µg/mL) or aspirin (1 mM) when indicated. One hour later, cells were stimulated with PAR2-AP (50 µM) or FVIIa (20 nM, 50 nM or 100 nM) for 60 minutes. Next, cells were treated with cisplatin (CDDP, 20 µM) for 48 h. On the indicated day, the cells were trypsinized, centrifuged and washed twice with phosphate-buffered saline (PBS). The cells were then stained with Nicoletti buffer (0.1% sodium citrate, 0.1% NP-40, 200 μg/ml RNase and 50 μg/ml propidium iodide). Doublets and debris were identified and excluded. Analysis of the DNA content was performed by collecting 30,000 events using the BD Accuri C6 flow cytometer (BD Biosciences, CA, USA). Cells with fragmented DNA (sub-G1 peak) were considered apoptotic cells.

### Transcriptome data obtained from TCGA

The mRNA data were extracted from The Cancer Genome Atlas (TCGA, http://cancergenome.nih.gov/), which is based on the transcriptome data obtained by RNA-seq V2. The subset of TCGA data included the RNA sequencing of 309 samples from cervical squamous cell carcinoma and endocervical adenocarcinoma.

### Overall survival study using the open access database cBioportal

cBioPortal for Cancer Genomics provided visualization, analysis, and the ability to download large-scale cancer genomics data sets [56, 57]. Cervical squamous cell carcinoma and endocervical adenocarcinoma (TCGA, provisional; *n* = 290) were selected to observe the relationship between the upregulation of *EGFR*, TF (*F3* gene), PAR1 (*F2R* gene), PAR2 (*F2RL1* gene) and COX2 (*PTGS2* gene) with overall survival. To define which tumors present the upregulation of the investigated mRNAs, the threshold was set in the z-score ± 1.5 SD. The z-scores for mRNA expression were determined for each sample by comparing a gene's mRNA expression to the distribution in a reference population that represents typical expression for the gene. If expression data were available for normal adjacent tissues, those data were used as the reference population; otherwise, expression values of all tumors that were diploid for the gene in question in the study were used [56, 57].

### Statistical analysis

The results were expressed as mean ± SD. Statistical analyses were performed using GraphPad Prism 5 (GraphPad Software, CA, USA). One-way analysis of variance (ANOVA) followed by Tukey’s post-test was used for the comparison between the test groups. If only two groups were compared, the unpaired 2-tailed Student’s *t*-test was applied. The correlation between gene expression measurements across all tumor tissues (TCGA) was analyzed by Spearman’s rank correlation. The Kaplan–Meier method was used to construct survival curves, and statistical significance between these curves was determined by the log-rank test. Differences were considered significant when *P <* 0.05.

## SUPPLEMENTARY MATERIALS FIGURES



## Abbreviations

ATP7A: ATPase copper transporting alpha
ATP7B: ATPase copper transporting beta
BAX: Bcl-2-associated X protein
CDDP: cis-diamminedichloroplatinum
COX: cyclooxygenase
EGFR: epidermal growth factor receptor
ERCC1: excision repair cross-complementation group 1
ERK: extracellular signal-regulated kinase
FVIIa: activated factor VII
GAPDH: glyceraldehyde 3-phosphate dehydrogenase
GPCR: G protein-coupled receptor
HPV: human papillomavirus
HSC70: heat shock cognate protein 70
MAPK: mitogen-activated protein kinase
PAR: protease-activated receptor
PAR1-AP: PAR1-specific agonist peptide
PAR2-AP: PAR2-specific agonist peptide
PARP: poly (ADP-ribose) polymerase
PGE2: prostaglandin E2
PPP: platelet-poor plasma
TCGA: the cancer genome atlas
TF: tissue factor
TPM: transcripts per million
XIAP: X-linked inhibitor of apoptosis protein
