# Supplementary material for: Positive crosstalk between EGFR and the TF-PAR2 pathway mediates resistance to cisplatin and poor survival in cervical cancer
Source: Oncotarget. 2018 Jul 17;9(55):30594–609. doi: 10.18632/oncotarget.25748 (PMC6078136; doi:10.18632/oncotarget.25748)
Supplement: Supplementary file 1 [file oncotarget-09-30594-s001.pdf]

Positive crosstalk between EGFR and the TF-PAR2 pathway mediates resistance to cisplatin and poor survival in cervical cancer

SUPPLEMENTARY MATERIALS

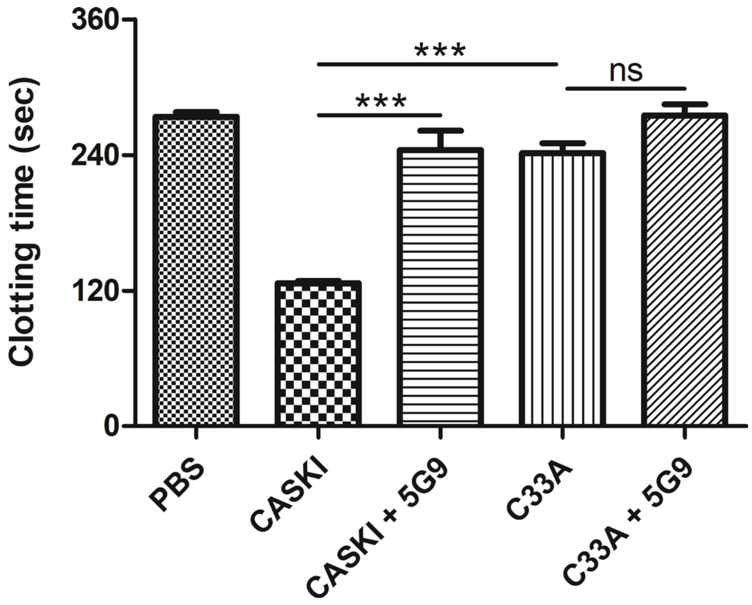

Supplementary Figure 1: CASKI cells induce plasma coagulation more potently than C33A cells, in a TF-dependent manner. CASKI and C33A cells (1 × 10^5 cells/mL) were pre-incubated with 50 µg/mL of a neutralizing anti-TF antibody (TF8-5G9; concentration relative to the final 200 µL volume in the coagulometer) for 15 min at room temperature. Cells were then added to platelet-poor plasma and clotting reaction was started with $CaCl_2$, and the control group (PBS) was evaluated without cells. Values represent mean + SD of three independent experiments; ns: not significant, $^{***}P < 0.001$ (one-way ANOVA).

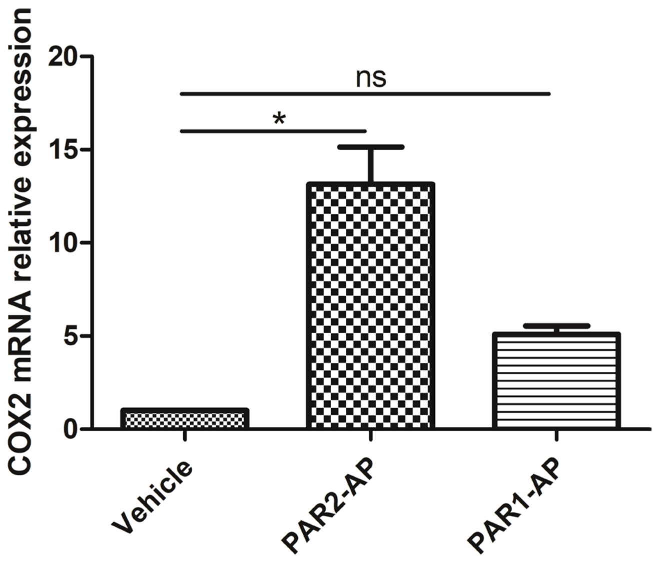

Supplementary Figure 2: PAR1 activation, unlike PAR2, does not increase COX2 expression in CASKI cells. Cells were starved for 16 h and were treated with PAR1-AP (50 μM) or PAR2-AP (50 μM). After 1.5 h, mRNA was converted into cDNA. COX2 expression was performed by qPCR and *GAPDH* was used as a reference gene. The relative expression level of mRNA was calculated using the ΔΔCT method. Values represent mean + SD of two independent experiments; ns: not significant, $^{*}P < 0.05$ (one-way ANOVA).

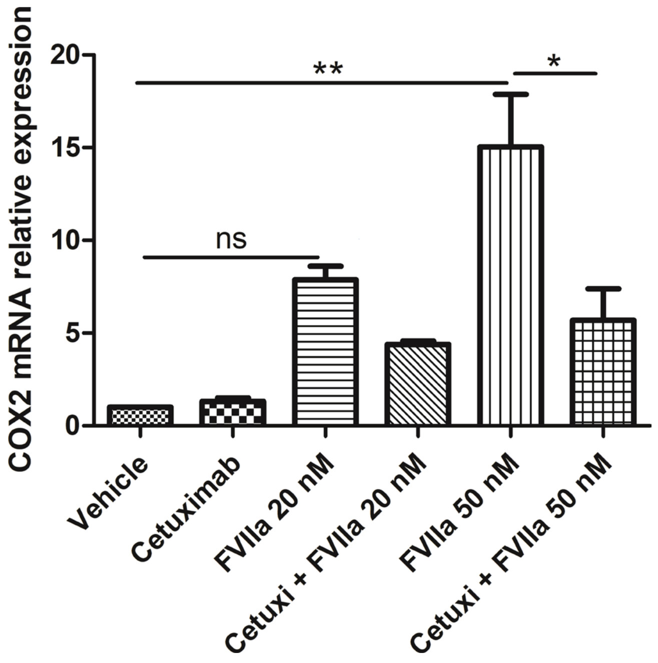

Supplementary Figure 3: FVIIa upregulates cyclooxygenase-2 (COX2) expression through an EGFR-dependent mechanism in CASKI cells. Cells were starved for 16 h and were treated with cetuximab (100 μg/mL). One hour later, cells were stimulated with FVIIa (20 nM or 50 nM). After 1.5 h, total RNA was extracted, and mRNA was converted into cDNA. The expression of COX2 (*PTGS2* gene) and *GAPDH* (reference gene) was performed by qPCR. The relative expression level of mRNA was calculated using the ΔΔCT method. Values represent mean + SD of two independent experiments; ns: not significant, $^{*}P < 0.05$, $^{**}P < 0.01$ (one-way ANOVA).
